# DNMT1 SNPs (rs2114724 and rs2228611) associated with positive symptoms in Chinese patients with schizophrenia

**DOI:** 10.1186/s12991-023-00466-x

**Published:** 2023-10-13

**Authors:** Junjiao Ping, Jing Wan, Caiying Huang, Jinming Yu, Jiali Luo, Zhiqiang Xing, Xingguang Luo, Baoguo Du, Tingyun Jiang, Jie Zhang

**Affiliations:** 1 Department of Psychiatry, The Third People’s Hospital, Zhongshan, 528451 Guangdong People’s Republic of China; 2 Joint Laboratory of Psychiatric Genetic Research, The Third People’s Hospital, Zhongshan, 528451 Guangdong People’s Republic of China; 3 Department of Clinical Psychology, The Third People’s Hospital, Zhongshan, 528451 Guangdong People’s Republic of China; 4 Department of Early Intervention, The Third People’s Hospital, Zhongshan, 528451 Guangdong People’s Republic of China; 5 Department of Psychiatry, Yale University School of Medicine, New Haven, CT 06510 USA; 6 Department of Psychiatry, Gannan Medical University, Ganzhou, 341000 Jiangxi People’s Republic of China

**Keywords:** Schizophrenia, Single nucleotide polymorphism (SNP), DNA methyltransferases, Symptoms

## Abstract

**Objective:**

Schizophrenia is a serious mental disorder with complex clinical manifestations, while its pathophysiological mechanism is not fully understood. Accumulated evidence suggested the alteration in epigenetic pathway was associated with clinical features and brain dysfunctions in schizophrenia. DNA methyltransferases (DNMTs), a key enzyme for DNA methylation, are related to the development of schizophrenia, whereas the current research evidence is not sufficient. The aim of study was to explore the effects of gene polymorphisms of DNMTs on the susceptibility and symptoms of schizophrenia.

**Methods:**

The study was case–control study that designed and employed the Diagnostic and Statistical Manual of Mental Disorders-Fifth Edition (DSM-5) as the diagnostic standard. 134 hospitalized patients with schizophrenia in the Third People's Hospital of Zhongshan City from January 2018 to April 2020 (Case group) as well as 64 healthy controls (Control group) from the same region were involved. Single nucleotide polymorphisms (SNPs) of DNMT1 genes (r s2114724 and rs 2228611) and DNMT3B genes (rs 2424932, rs 1569686, rs 6119954 and rs 2424908) were determined with massARRAY. Linkage disequilibrium analysis and haplotype analysis were performed, and genotype and allele frequencies were compared. The Hardy–Weinberg equilibrium was tested by the Chi-square test in SPSS software (version 20.0, SPSS Inc., USA). The severity of clinical symptoms was assessed by the Positive and Negative Syndrome Scale (PANSS). The correlation between DNMT1 genes (rs 2114724 and rs 2228611) and DNMT3B genes (rs2424932, rs1569686, rs6119954 and rs2424908) and clinical features was analyzed.

**Results:**

There were no significant differences in genotype, allele frequency and haplotype of DNMT1 genes (rs 2114724 and rs 2228611) and DNMT3B genes (rs 2424932, rs 1569686, rs 6119954 and rs 2424908) between the case and healthy control group. There were significant differences in the PANSS total positive symptom scores, P3 (hallucinatory behavior), P6 (suspicious/persecution), G7 (motor retardation), and G15 (preoccupation) in patients with different DNMT1 gene rs 2114724 and rs 2228611 genotypes. The linkage disequilibrium analysis of gene polymorphic loci revealed that rs 2114724–rs 2228611 was complete linkage disequilibrium, and rs 1569686–rs 2424908, rs 2424932–rs 1569696 and rs 2424932–rs 2424908 were strongly linkage disequilibrium.

**Conclusion:**

The polymorphisms alteration in genetic pathway may be associated with development of specific clinical features in schizophrenia.

## Introduction

Schizophrenia is a chronic and debilitating mental disorder affecting more than 20 million individuals worldwide, with significant socioeconomic consequences [1, 2]. One of the main features of schizophrenia is the dissociation of thoughts, concepts, identities, and emotions [3–6], associated with dysregulated neural pathways in the brain that lead to positive and negative symptoms [4, 6, 7]. The etiology of schizophrenia is multifactorial, involving genetic predisposition, environmental factors, and epigenetic modifications [6]. It is estimated that schizophrenia has a heritability range of 79–81% [8]. Genetic risk loci have been identified in non-coding areas such as promoters and introns, indicating that gene regulation is crucial for the onset of psychotic disorders [9]. In the case of schizophrenia, research has identified a number of genetic risk factors that are associated with altered gene expression patterns [10–12], which may be influenced by epigenetic modifications [9]. Twin studies have also provided evidence that epigenetic factors may be involved in the development of schizophrenia [13].

Epigenetic modifications, mainly DNA methylation or histone modifications, are involved in gene regulation. Among these, DNA methylation is the most extensively investigated [14]. DNA methylation occurs through the covalent binding of methyl groups to cytosine-guanine dinucleotides (CpG) [15]. Several genes, including *GAD1* [16], *COMT* [17], *RELN* [18], *BDNF* [19], *CHRNA7* [20], *SOX10* [21], *EGR1* [22], have been identified as potential candidates for DNA methylation in schizophrenic patients. DNA methylation is established and maintained by methyltransferases, such as DNMT1, DNMT3A, and DNMT3B [23]. *DNMTs* were first recognized as stable repressive marks, which are important mechanisms for gene silencing during progression and the cycle of life [24]. It has been suggested that patients with schizophrenia may have DNMT gene dysfunction.

Studies have shown that *DNMTs* are essential for the generation of mature, functioning germ cells, and appropriate embryonic development [25, 26]. Abnormal DNA methylation patterns have been linked to an increased risk of certain human diseases in individuals with a hereditary predisposition [27]. Genetic variations, such as single nucleotide polymorphisms (SNPs), have been strongly associated with schizophrenia in several family studies [28, 29]. Recent case–control studies indicated a significant association between minor alleles of *DNMT1* (rs2114724 and rs2228611), *DNMT3B* (rs2424932 and rs1569686) and susceptibility of schizophrenia, while it is rare [30].

Previous studies have focused on the three active DNMT enzymes: DNMT1, DNMT3A, and DNMT3B. Some investigations have linked schizophrenia to polymorphisms within these enzymes, but there is still considerable ambiguity regarding the relationship between these polymorphisms and the clinical features of schizophrenia. DNMT SNPs prevalence in southern Chinese populations has not been reported yet. Therefore, the aim of the present study was to explore the effects of DNMT gene polymorphisms on schizophrenia susceptibility and symptoms in this population.

## Materials and methods

### Patient and control samples

Patients or their legal guardians provided informed consent after receiving authorization from the ethics committee of Zhongshan Third People’s Hospital for this study. The investigation focused on patients with schizophrenia who were treated at the hospital between 2018 and 2020. Participants were included if they met the following criteria: a diagnosis of schizophrenia as per the Diagnostic and Statistical Manual of Mental Disorders, Fifth Edition, made by two attending physicians with consistent training, age between 16 and 60 years, and Han ethnicity. The study excluded patients with diabetes, hypertension, cancer, and other nervous system or mental diseases. However, other mental disorders were not excluded.

The control group at Zhongshan City’s Third People’s Hospital consisted of physical exam mentally healthy patients, employees, nurses, and volunteers. To be included in the control group, subjects had to meet the following criteria: no history of mental illness, no family history of mental illness, pass a physical exam, Han ethnicity, and between 18 and 60 years of age after psychological screening. The control group excluded adopted or single-parent children with unknown family histories, as well as individuals with serious somatic disorders such as diabetes, hypertension, or cancer.

### Methods

The experimental methods used in this research included the collection of general data and clinical information, evaluation of psychotic symptoms, genomic DNA extraction, primer creation, PCR amplification, alkaline phosphatase treatment, single-base extension reaction, resin purification, chip sampling, mass spectrometric detection, and data analysis, which were based on past studies [31, 32].

The collected data were sorted and evaluated using the statistical program SPSS 20.0 (IBM, Armonk, NY, USA). The analysis of variance was performed, and results were presented as mean and standard deviation since the data exhibited normal distribution. Nonparametric statistical approaches were applied to non-normal data, and the results were expressed numerically as the median value (upper quartile, lower quartile). The genotype frequency was analyzed using the Chi-square test. regarding as categorical data. Hardy–Weinberg equilibrium was examined using SPSS 20.0 Chi-square test, and genotype and allele frequencies were compared between the schizophrenia and control groups. The linkage disequilibrium (LD) and pairwise LD coefficients in DNMT1 and DNMT3B were implemented with Haploview 4.2. ORs and confidence intervals (CIs) were computed for 95% of the data, and G*Power 3.1 was used for power analysis.

## Results

### Test of Hardy–Weinberg equilibrium for *DNMT1* and *DNMT3B*

We performed a Hardy–Weinberg equilibrium analysis on the genotype data of 134 individuals with schizophrenia, focusing on six *DNMT* gene polymorphisms (rs2114724, rs2228611, rs2424932, rs1569686, rs6119954, and rs2424908). The results, presented in Tables 1 and 2, indicate that the genotype frequencies at these loci did not deviate significantly from the predicted population frequencies (*p* > 0.05). No outliers were identified as the cause of these results.

**Table 1:** Hardy–Weinberg equilibrium test for *DNMT1* genotypes

Locus	Group (*n*)	Genotype	Frequency (%)	*X*^2^	*P*
rs2114724	Schizophrenia group (134)	CC	57 (42.5)	0.84	0.66
		CT	57 (42.5)		
		TT	20 (15.0)		
	Control group (64)	CC	30 (46.9)	0.06	0.97
		CT	27 (42.2)		
		TT	7 (10.9)		
rs2228611	Schizophrenia group (134)	CC	57 (42.5)	0.84	0.66
		CT	57 (42.5)		
		TT	20 (15.0)		
	Control group (64)	CC	30 (46.9)	0.06	0.97
		CT	27 (42.2)		
		TT	7 (10.9)

**Table 2:** Hardy–Weinberg equilibrium test for *DNMT3B* genotypes

Locus	Group (*n*)	Genotype	Frequency (%)	*X*^2^	*P*
rs1569686	Schizophrenia group (134)	GG	3 (2.2)	17.8	0.00
		GT	9 (6.7)		
		TT	122 (91.1)		
	Control group (64)	GG	1 (1.6)	1.71	0.43
		GT	7 (10.9)		
		TT	56 (87.5)		
rs2424908	Schizophrenia group (134)	CC	23 (17.2)	0.78	0.68
		CT	59 (44.0)		
		TT	52 (38.8)		
	Control group (64)	CC	9 (14.1)	0.16	0.92
		CT	32 (50.0)		
		TT	23 (35.9)		
rs2424932	Schizophrenia group (134)	AA	0 (0)	0.29	0.86
		AG	12 (8.9)		
		GG	122 (91.0)		
	Control group (64)	AA	0 (0)	0.28	0.87
		AG	8 (12.5)		
		GG	56 (87.5)		
rs6119954	Schizophrenia group (134)	AA	8 (6.0)	0.54	0.76
		AG	56 (41.8)		
		GG	70 (52.2)		
	Control group (64)	AA	3 (4.7)	0.94	0.62
		AG	28 (43.8)		
		GG	33 (51.5)		

### Results of genotype and allelic analysis for *DNMT1* and *DNMT3B*

Tables 3 and 4 demonstrate that there were no significant differences in genotype frequencies between the two groups at the *DNMT1* gene loci rs2114724 and rs2228611 (*p* > 0.05). Likewise, no significant differences were observed in the genotype frequencies between the two groups at the *DNMT3B* gene loci rs2424932, rs1569686, rs6119954, and rs2424908 (*p* > 0.05).

**Table 3:** Comparison of *DNMT1* rs2114724 and rs2228611 genotype frequencies between the two groups

Group	Cases	rs2114724			rs2228611		
		CC	CT	TT	CC	CT	TT
Schizophrenia group	134	57	57	20	57	57	20
Control group	64	30	27	7	30	27	7
*p*	*/*	0.708			0.692

**Table 4:** Comparison of *DNMT3B* rs1569686, rs2424908, rs2424932 and rs6119954 genotype frequencies between the two groups

Group	Cases	rs1569686			rs2424908			rs2424932			rs6119954		
		GG	GT	TT	CC	CT	TT	AA	AG	GG	AA	AG	GG
Schizophrenia group	134	3	9	122	23	59	52	0	12	122	8	56	70
Control group	64	1	7	56	9	32	23	0	8	56	3	28	33
*p*	*/*	0.573			0.709			0.436			0.918		

Table 5 displays no significant differences in the frequency of the rs2114724, rs2228611, rs1569686, rs2424908, rs2424932, and rs6119954 alleles between the two groups (*p* > 0.05).

**Table 5:** Comparison of rs2114724, rs2228611, rs1569686, rs2424908, rs2424932 and rs6119954 allele frequencies between the two groups

Group	Cases	rs2114724		rs2228611		rs1569686		rs2424908		rs2424932		rs6119954	
		C	T	C	T	G	T	C	T	A	G	A	G
Schizophrenia group	134	171	97	171	97	15	253	105	163	12	256	72	196
Control group	64	87	41	87	41	9	119	50	78	8	120	34	94
*p*	*/*	0.416		0.416		0.576		0.902		0.451		0.949	

### Linkage disequilibrium analysis for *DNMT1* and *DNMT3B*

**Fig. 1:**
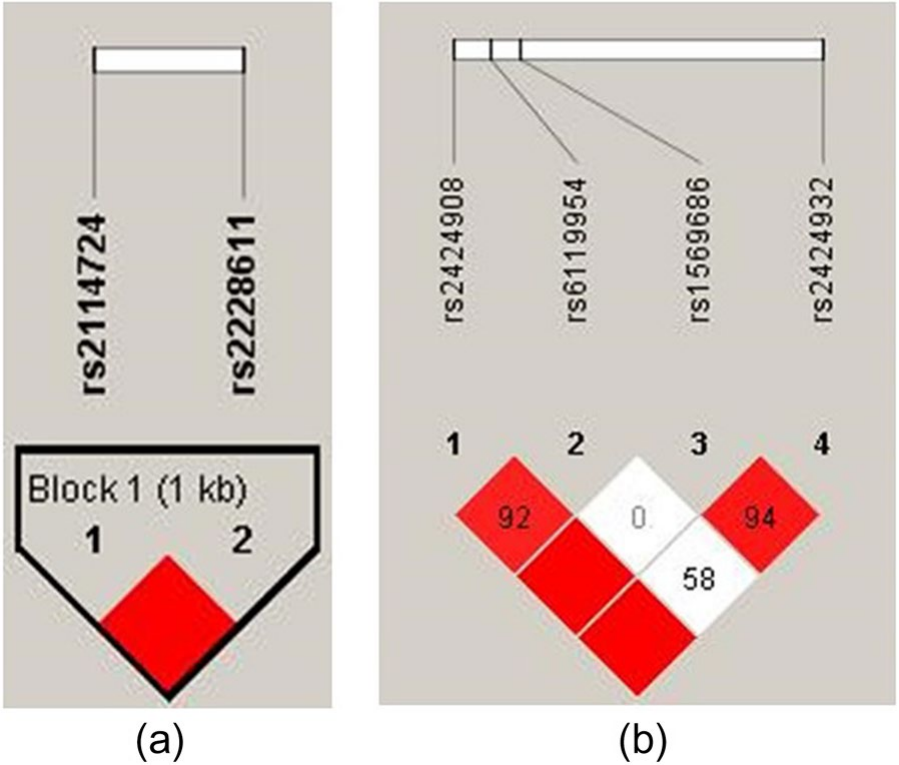
Linkage disequilibrium (LD) pattern of DNMT1 (**a**) and DNMT3B (**b**) SNPs from Haploview analysis. The D′ values for LD are represented by the numbers inside the boxes; the miss numbers were 100. The strength of the LD between pairwise pairings of SNPs is shown by the areas that are shaded in the LD map (white represent low LD; red represent high LD)

Genotyping for two SNPs in *DNMT1* and four SNPs in *DNMT3B* was successful, and Fig. 1 illustrates their linkage disequilibrium (LD). The two SNPs in *DNMT1* formed a block within 25 kb (chr19: 10154572-10156401), contrasting the four SNPs in *DNMT3B*. Due to *DNMT1's* strong linkage, tagged SNPs were run in Haploview.

### Haplotypes of the SNPs in *DNMT1* and *DNMT3B*

We examined the haplotype distribution of *DNMT1* (rs2114724 and rs2228611) and *DNMT3B* (rs1569686, rs2424908, rs2424932, and rs6119954) in both the schizophrenia and control groups. Haplotypes with a frequency of at least 3% were selected for analysis to investigate their association with schizophrenia. Two distinct *DNMT1* haplotypes were identified, with the CT haplotype being the most common in both controls (68.0%) and patients (63.8%). The most common *DNMT3B* haplotype in both groups was GTGT (59.3% and 60.9%, respectively). The haplotype frequencies at gene loci rs2114724–rs2228611, rs2424932–rs1569686–rs6119954–rs2424908, and overall did not differ significantly between patients and controls (*p* > 0.05, Table 6).

**Table 6:** Analysis of haplotype distribution between the schizophrenia and control groups

Genes	Haplotype	Cases, *n* (frequency)	Controls, *n* (frequency)	*p*	OR (95% CI)
*DNMT1*	CT	171 (0.638)	87 (0.680)	0.416	0.831 (0.531, 1.299)
	TT	97 (0.362)	41 (0.320)	0.416	1.204 (0.770, 1.882)
*DNMT3B*	AGGC	11 (0.041)	8 (0.062)	0.382	0.660 (0.259, 1.684)
	GTAC	65 (0.245)	33 (0.258)	0.830	0.948 (0.583, 1.542)
	GTGC	25 (0.095)	8 (0.062)	0.296	1.551 (0.678, 3.552)
	GTGT	159 (0.593)	78 (0.609)	0.987	0.997 (0.644, 1.541)

### Analysis of the association between genotypes and clinical symptoms of schizophrenia

Table 7 displays the associations between *DNMT1* (rs2114724 and rs2228611) and *DNMT3B* gene (rs1569686, rs2424908, rs2424932, and rs6119954) and clinical mental symptoms of schizophrenia. Statistically significant differences in PANSS overall positive symptom scores, P3 (hallucinatory behavior), P6 (suspicious/persecution), G7 (motor retardation), and G15 (preoccupation) were observed when comparing patients with different *DNMT1* gene rs2114724 and rs2228611 genotypes. Moreover, significant differences were found in PANSS general symptom scores G1 (somatic concern), G5 (mannerisms and posturing), and G8 (uncooperativeness) among patients with different *DNMT3B* gene rs6119954 genotypes (*p* < 0.05). Additionally, significant differences were noted in the PANSS general symptom score G10 (disorientation) among individuals with different *DNMT3B* gene rs2424908 genotypes (*p* < 0.05).

**Table 7:** Relationships between different DNMT1 and DNMT3B genotypes and PANSS scores in schizophrenia group

SNP		**rs2114724**			**rs2228611**			**rs1569686**			**rs2424908**			**rs2424932**		**rs6119954**		
Genotype (n)		CC (57)	CT (57)	TT (20)	CC (57)	CT (57)	TT (20)	GG (3)	GT (9)	TT (122)	CC (23)	CT (59)	TT (52)	AG (12)	GG (122)	AA (8)	AG (56)	GG (70)
P1 (delusions)	*X*^2^ (*P)*	6.201 (0.045)			6.201 (0.045)			3.054 (0.217)			2.183 (0.336)			1.078 (0.281)		1.664 (0.435)		
P2 (conceptual disorganization)	*X*^2^ (*P)*	0.715 (0.699)			0.715 (0.699)			0.158 (0.924)			0.542 (0.763)			0.044 (0.965)		1.555 (0.460)		
P3 (hallucinatory behavior)	*X*^2^ (*P)*	**7.851 (0.020)**			**7.851 (0.020)**			5.685 (0.058)			0.052 (0.975)			1.878 (0.060)		0.020 (0.990)		
P4 (excitement)	*X*^2^ (*P)*	0.300 (0.861)			0.300 (0.861)			0.888 (0.641)			4.028 (0.133)			0.894 (0.371)		3.662 (0.160)		
P5 (grandiosity)	*X2* (*P)*	1.264 (0.532)			1.264 (0.532)			0.918 (0.632)			0.219 (0.896)			0.315 (0.753)		5.937 (0.051)		
P6 (suspiciousness/persecution)	*X*^2^ (*P)*	**10.387 (0.006)**			**10.387 (0.006)**			2.659 (0.265)			0.209 (0.901)			1.292 (0.196)		0.431 (0.806)		
P7 (hostility)	*X*^2^ (*P)*	1.954 (0.376)			1.954 (0.376)			2.142 (0.343)			1.062 (0.588)			0.203 (0.839)		1.474 (0.478)		
Total of positive symptoms	*X*^2^ (*P)*	**8.471 (0.014)**			**8.471 (0.014)**			1.995 (0.369)			0.310 (0.856)			0.967 (0.334)		0.610 (0.737)		
N1 (blunted affect)	*X*^2^ (*P)*	0.877 (0.645)			0.877 (0.645)			1.579 (0.454)			2.963 (0.227)			0.723 (0.470)		2.963 (0.227)		
N2 (emotional withdrawal)	*X*^2^ (*P)*	2.692 (0.260)			2.692 (0.260)			2.830 (0.243)			3.709 (0.157)			0.208 (0.837)		2.899 (0.235)		
N3 (poor rapport)	*X*^2^ (*P)*	1.376 (0.502)			1.376 (0.502)			0.863 (0.649)			1.382 (0.501)			0.790 (0.430)		1.337 (0.512)		
N4 (passive/apathetic social withdrawal)	*X*^2^ (*P)*	1.886 (0.389)			1.886 (0.389)			3.987 (0.136)			1.640 (0.440)			0.487 (0.627)		0.085 (0.958)		
N5 (difficulty in abstract thinking)	*X*^2^ (*P)*	1.329 (0.515)			1.329 (0.515)			0.736 (0.692)			2.147 (0.342)			0.520 (0.603)		1.537 (0.464)		
N6 (lack of spontaneity and flow of conversation)	*X*^2^ (*P)*	0.720 (0.698)			0.720 (0.698)			0.019 (0.990)			0.500 (0.779)			0.138 (0.890)		0.037 (0.982)		
N7 (stereotyped thinking)	*X*^2^ (*P)*	4.471 (0.107)			4.471 (0.107)			3.009 (0.222)			0.607 (0.738)			0.554 (0.580)		2.333 (0.311)		
Total of negative symptoms	*X*^2^ (*P)*	2.359 (0.307)			2.359 (0.307)			1.404 (0.496)			2.412 (0.299)			0.077 (0.938)		2.287 (0.319)		
G1 (somatic concern)	*X*^2^ (*P)*	1.253 (0.534)			1.253 (0.534)			3.377 (0.185)			3.283 (0.194)			0.331 (0.740)		**7.148 (0.028)**		
G2 (anxiety)	*X*^2^ (*P)*	5.256 (0.072)			5.256 (0.072)			1.602 (0.449)			1.266 (0.531)			0.662 (0.508)		0.155 (0.925)		
G3 (guilt feeling)	*X*^2^ (*P)*	5.584 (0.061)			5.584 (0.061)			1.275 (0.529)			0.008 (0.996)			0.626 (0.531)		2.286 (0.319)		
G4 (tension)	*X*^2^ (*P)*	2.819 (0.244)			2.819 (0.244)			2.708 (0.258)			0.810 (0.667)			0.184 (0.854)		0.004 (0.998)		
G5 (mannerisms and posturing)	*X*^2^ (*P)*	0.300 (0.861)			0.300 (0.861)			0.539 (0.764)			1.022 (0.600)			0.583 (0.560)		**6.077 (0.048)**		
G6 (depression)	*X*^2^ (*P)*	4.176 (0.124)			4.176 (0.124)			0.276 (0.871)			1.539 (0.463)			0.217 (0.828)		1.688 (0.430)		
G7 (motor retardation)	*X*^2^ (*P)*	**6.656 (0.036)**			**6.656 (0.036)**			1.690 (0.430)			0.307 (0.857)			0.276 (0.782)		1.384 (0.500)		
G8 (uncooperativeness)	*X*^2^ (*P)*	3.692 (0.158)			3.692 (0.158)			1.840 (0.399)			0.127 (0.938)			0.068 (0.946)		**6.923 (0.031)**		
G9 (unusual thought content)	*X*^2^ (*P)*	5.050 (0.080)			5.050 (0.080)			0.839 (0.657)			1.691 (0.429)			0.283 (0.777)		1.464 (0.481)		
G10 (disorientation)	*X*^2^ (*P)*	0.371 (0.831)			0.371 (0.831)			2.825 (0.243)			**6.927 (0.031)**			0.433 (0.665)		3.325 (0.190)		
G11 (poor attention)	*X*^2^ (*P)*	6.003 (0.050)			6.003 (0.050)			2.197 (0.333)			0.232 (0.890)			0.222 (0.825)		1.853 (0.396)		
G12 (lack of judgment and insight)	*X*^2^ (*P)*	2.399 (0.301)			2.399 (0.301)			2.151 (0.341)			1.135 (0.567)			0.463 (0.643)		3.279 (0.194)		
G13 (disturbance of volition)	*X*^2^ (*P)*	3.959 (0.138)			3.959 (0.138)			5.174 (0.075)			1.434 (0.488)			0.634 (0.526)		0.117 (0.943)		
G14 (poor impulse control)	*X*^2^ (*P)*	1.365 (0.505)			1.365 (0.505)			0.354 (0.838)			3.256 (0.196)			0.036 (0.971)		0.210 (0.900)		
G15 (preoccupation)	*X*^2^ (*P)*	**10.222 (0.006)**			**10.222 (0.006)**			1.372 (0.504)			0.236 (0.888)			1.065 (0.287)		0.649 (0.723)		
G16 (active social avoidance)	*X*^2^ (*P)*	1.724 (0.422)			1.724 (0.422)			1.199 (0.549)			1.340 (0.512)			0.420 (0.675)		1.087 (0.581)		
Total of general symptom	*X*^2^ (*P)*	5.710 (0.058)			5.710 (0.058)			1.283 (0.527)			0.027 (0.986)			0.191 (0.849)		1.415 (0.493)		
Positive symptoms	*X*^2^ (*P)*	**7.938 (0.019)**			**7.938 (0.019)**			2.579 (0.275)			2.389 (0.303)			1.245 (0.213)		1.617 (0.446)		
Negative symptoms	*X*^2^ (*P)*	1.493 (0.474)			1.493 (0.474)			1.924 (0.382)			3.379 (0.185)			0.066 (0.948)		1.888 (0.389)		
Cognitive impairment	*X*^2^ (*P)*	0.951 (0.621)			0.951 (0.621)			0.525 (0.769)			0.959 (0.619)			0.180 (0.857)		2.162 (0.339)		
Excited/hostile	*X*^2^ (*P)*	2.261 (0.322)			2.261 (0.322)			0.937 (0.626)			1.646 (0.439)			0.525 (0.600)		3.353 (0.187)		
Anxiety/depression	*X*^2^ (*P)*	4.999 (0.082)			4.999 (0.082)			0.779 (0.677)			1.293 (0.524)			0.303 (0.762)		0.891 (0.640)		
PANSS (total)	*X*^2^ (*P)*	5.368 (0.068)			5.368 (0.068)			0.683 (0.711)			0.953 (0.621)			0.030 (0.976)		2.158 (0.340)		

The bold values mean the positive association between loci frequence of specific SNP with the type of clinical symptoms

### Evaluation of statistical power

G*Power calculated power. This sample had 92.372% power to identify a meaningful relationship (*α* < 0.05) with an effect size index of 0.5.

## Discussion

Accumulating evidence suggests that the development of schizophrenia is influenced by the interaction of heredity and environmental factors [33]. Epigenetic mechanisms, such as DNA methylation with DNA methyltransferases (DNMTs), are crucial for the effect of environmental factors on genetic susceptible. In the dynamic procedure methylation, the DNMT1 is responsible majorly for maintaining methylation during cell division [34, 35].

This study investigated the genotype and allele distribution of *DNMT1* (rs2114724 and rs2228611) and *DNMT3B* (rs1569686, rs2424908, rs2424932, and rs6119954) in 134 patients with schizophrenia and 64 healthy controls. The results showed no statistical difference between the case and control groups, which contradicted to the few previous study in India population [36]. The study found that the TT genotype and T allele in DNMT1 rs2114724 and the AA genotype and A allele in *DNMT1* rs2228611 were associated with schizophrenia. However, there was no significant difference in genotype and allele distribution in *DNMT3B* gene (rs2424932, rs1569686, rs6119954, and rs2424908) between the case group and the control group. In line with study from population with shared ethical background [37], indicated that the genotype frequency and allelic frequency of *DNMT1* rs2114724 and rs2228611 were also correlated with schizophrenia. Few studies employing Chinese Han population reported similar findings with the current study. For instance, Zhai et al. [38] investigated the genotype and allele distribution of *DNMT1* rs2114724 and rs2228611 in a Chinese population and found no significant difference between the schizophrenic patients and healthy controls. Furthermore, Zhang Chen et al. [39] revealed that there was no statistical difference in genotype and allele distribution of *DNMT3B* rs2424908 between the schizophrenic case and control groups. Although the significant difference in genotype and allele distribution of *DNMT3B* rs6119954 was identified in the schizophrenic patients, which indicated dysfunction of DNMR3B. There are couple of reasons contribute to the discrepancies. Firstly, racial genetic background will affect on the difference in allelic frequency, according to the previous findings [38, 39]. Secondly, technology employed for identifying the genetic variation attribute partly to difference of observational results. Previous study indicated that low effective values for identifying genetic variation in promoter upstream regions with Methylated DNA Immunoprecipitation (MeDIP) comparing to microarray [40]. Finally, sample size in the literatures may contribute to variation in the findings [41].

This study used linkage disequilibrium analysis to identify an absolute linkage block between rs2114724 and rs2228611 in *DNMT1* and the four loci in *DNMT3B*. These loci were significantly correlated with positive symptoms of schizophrenia, including hallucinatory behavior, suspicion/persecution, motor retardation, and preoccupation. This is the first report linking rs2114724 and rs2228611 in *DNMT1* with positive symptoms of schizophrenia. *DNMT1* gene may affect the clinical symptoms of schizophrenia by regulating the expression of genes involved in the dopaminergic and GABAergic systems [41]. Cumulating evidence has suggested that DNMT1 as well as DNMT3 established and maintained dynamically the DNA methylation in dopaminergic, GABAergic, glutamatergic, serotonergic pathways of neurotransmission, which response for regulating the clinical phenotypes [42–46].

Although this study provides new insights into the role of *DNMTs* in schizophrenia, limitations exist, and further research is necessary to validate the findings in larger samples and different populations. Future studies should also explore the specific regulatory mechanisms of DNA methylation in the development of schizophrenia.

## Data Availability

The original data presented in the study are available upon to enquiry from the corresponding authors.
